# Robotic resection of left renal vein with preservation of left kidney for leiomyosarcoma: Case report and review of the literature

**DOI:** 10.1016/j.ijscr.2024.109381

**Published:** 2024-02-15

**Authors:** Claudio Lodoli, Miriam Attalla El Halabieh, Francesco Santullo, Carlo Abatini, Valerio Gallotta, Fabio Pacelli

**Affiliations:** a Surgical Unit of Peritoneum and Retroperitoneum Surgery, Fondazione Policlinico Universitario A. Gemelli, IRCCS, Italy; b Division of Gynecologic Oncology, Department of Women and Children's Health, Fondazione Policlinico Universitario A. Gemelli, IRCCS, Italy

**Keywords:** Leiomyosarcoma, Soft tissue sarcomas, Minimally invasive surgery, Case report

## Abstract

**Introduction:**

Radical surgical resection with negative margins is the mainstay of treatment for retroperitoneal vascular leiomyosarcomas. Given the retroperitoneal location of these tumors, open surgery is, historically, the chosen surgical approach, however, it is burdened with high postoperative morbidity. In selected cases, the small dimension of the tumor and a favorable location, allow to perform a minimally invasive treatment.

**Presentation of case:**

A 67-year-old female patient with a diagnosis of a leiomyosarcoma arising from the left renal vein underwent a robotic resection of the left renal vein with preservation of the left kidney and a relative outflow trough the gonadal vessels. The patient was discharged on the fourth postoperative day without any complications and there was no tumor recurrence noted during the 24-month follow-up period.

**Discussion:**

Vascular retroperitoneal leiomyosarcomas are very rare tumors requiring a complete en bloc gross tumor resection in order to achieving microscopically negative margins on the vein of origin. Thanks to the preoperative histological diagnosis and radiological study of the neoplasm, it was possible to proceed to a highly personalized and minimally invasive treatment with respect of oncological criteria.

**Conclusion:**

In selected cases, a minimally invasive surgery of vascular leiomyosarcoma could be a feasible and safe treatment option.

## Highlights

• Vascular leiomyosarcoma is a very rare soft tissue sarcoma.
• Surgery with a complete primary resection and with negative margins is the gold standard treatment.
• Open surgery is burdened with high postoperative morbidity.
• In selected cases a minimally invasive surgery of vascular leiomyosarcoma could be pursued.

## 1 Introduction

Among soft tissue sarcomas, leiomyosarcoma (LMS) is one of the most common types, with an estimated incidence of <1/100000/year [1]. Vascular retroperitoneal LMS typically arises from the inferior vena cava (IVC) and, in rarer cases, from renal or gonadal veins [2]. Surgery with a complete primary resection and with negative margins is the gold standard treatment, as well as the most important prognostic factor for local recurrence and overall survival [3]. Given the retroperitoneal location of these tumors, open surgery is still nearly always required, but it is burdened by high post-operative complications. However, in selected cases of localized lesions, a minimally invasive approach could be pursued. This case report is in line with the SCARE criteria [4].

## 2 Case report

**Fig. 1:**
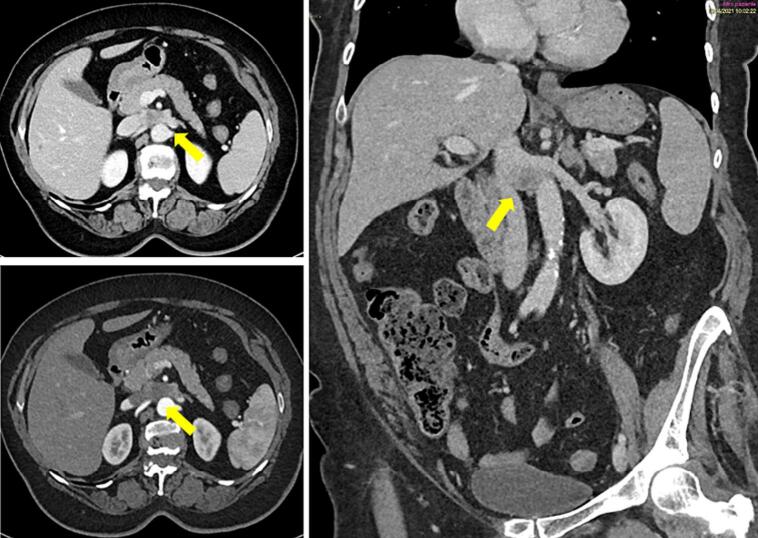
Preoperative CT-scan show a small lesion arising from the left renal vein.

**Fig. 2:**
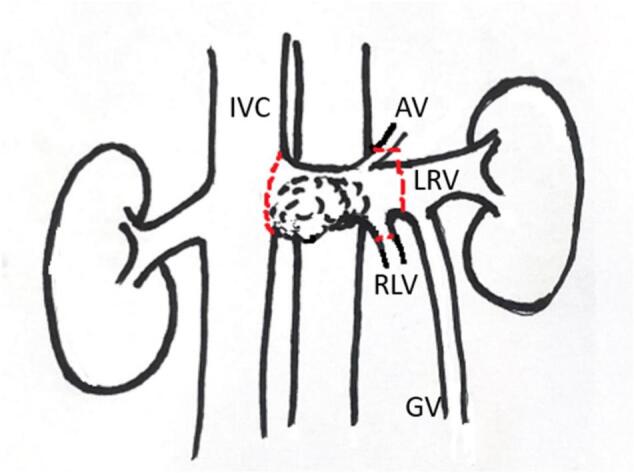
Vascular anatomy. Inferior Vena Cava (IVC), Adrenal Vein (AV), Left Renal Vein (LRV), Reno-Lumbar Vein (RLV), Gonadal Vein (GV).

**Fig. 3:**
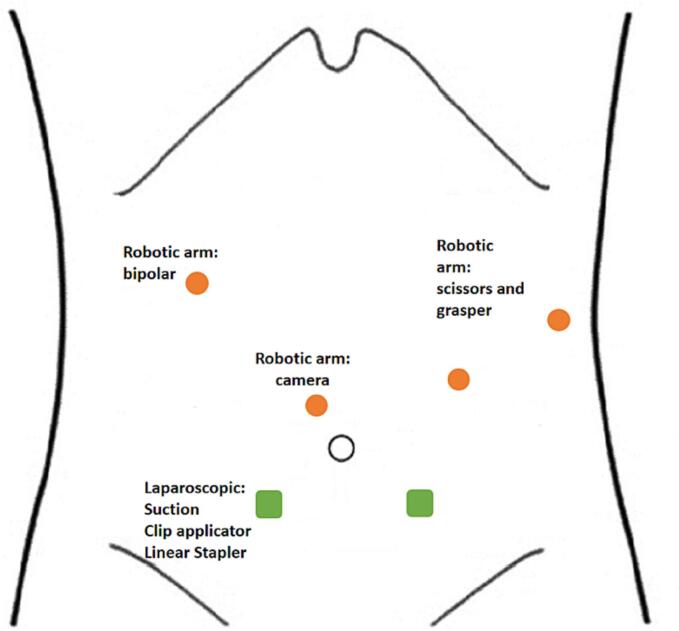
Robotic trocars placement.

**Fig. 4:**
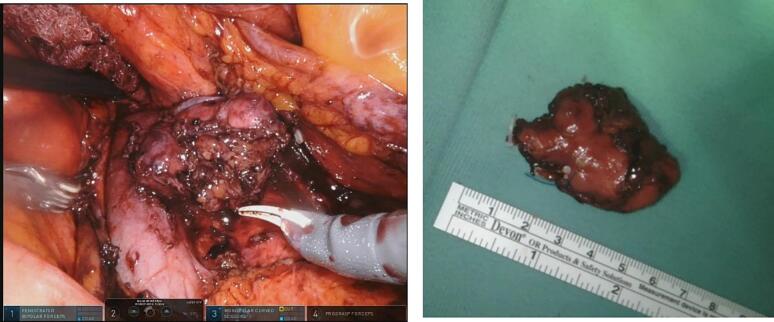
Intraoperative image of the tumor near to the IVC. Then, an image of the specimen extracted.

This is the case of a 67-year-old female patient with an accidental diagnosis of a retroperitoneal tumor arising from the left renal vein. The computerized tomography (CT) scan showed a solid hypodense lesion of 22 × 30 mm in the aortocaval space, arising from the left renal vein (Fig. 1). The preoperative histological report, obtained by endoscopic ultrasound-guided fine-needle aspiration biopsy, documented the neoplastic cells with focal high-grade atypia, thus suggesting muscle differentiation. The patient's medical history included obesity (body mass index 31), a prior case of breast cancer, C-section, as well as total hysterectomy and bilateral salpingo-oophorectomy with umbilical-pubic incision for endometrial cancer. She underwent a robotic resection of the left renal vein with preservation of the left kidney and a relative outflow trough the gonadal vessels (Fig. 2). Following an initial viscerolysis and laparoscopic incision of the mesentery root, the robotic docking was performed (Fig. 3). Then, the exposure of the aortocaval plane was performed by isolating the lesion, which was originating from the left renal vein, from the inferior vena cava and the infrarenal segment of the abdominal aorta. After locating the left renal artery, the plane between the anterior wall of the aorta and the left renal vein was developed. During the exposure of the lower margin of the left renal vein, a reno-lumbar vein was identified, isolated and sectioned using clips, as well as the left adrenal vein. Then, the section of the left renal vein, which was upstream of the left gonadal vein was performed using a 30 mm vascular linear stapler. The lesion was then detached from the aortic plane from the lateral to medial region. Subsequently, a partial section of the vena cava was tangentially carried out with a 30 mm vascular linear stapler and the specimen extracted (Fig. 4).

**Fig. 5:**
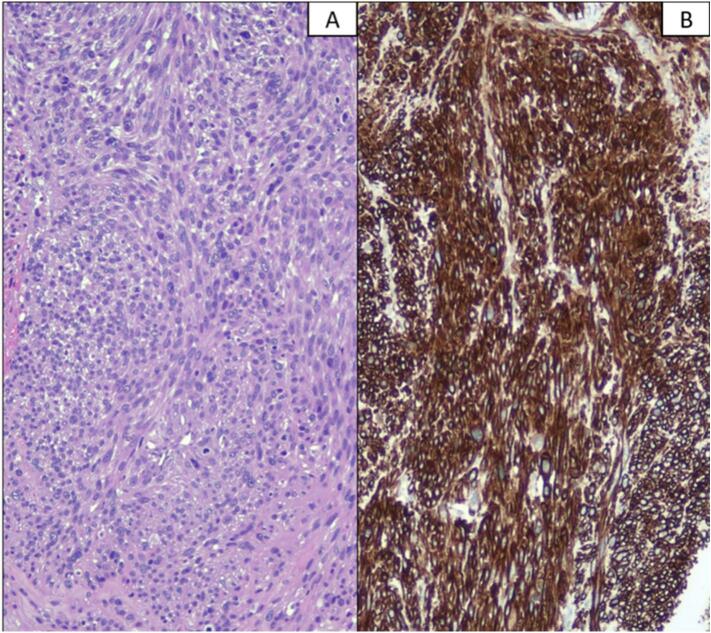
(A) Histologically, the neoplasm was composed of spindle cells with eosinophilic cytoplasm and sigar-shaped nuclei. The neoplastic cells showed a moderate grade of atypia, and necrosis was absent. The cells were positive for Caldesmon. (B) Smooth muscle actin and desmin.

On the third postoperative day, the patient underwent renal ultrasonography which documented the left renal artery and vein patent with traces that were within the limits. Renal function was normal. The patient was discharged on the fourth postoperative day without any complications. The pathology report showed a left renal vein leiomyosarcoma (4 × 2.2 × 2 cm) grade 2 s. FNCLCC (Fig. 5), with inferior vena cava (IVC) and left renal vein negative margins. There was no tumor recurrence noted during the 24-month follow-up period.

## 3 Discussion

Vascular LMS are rare extraperitoneal tumors, characterized by smooth muscle differentiation. The most common retroperitoneal site of origin is the inferior vena cava (IVC), which accounts for >50 % of cases. The rest of the case of vascular LMS develop from other vessels [5].

Radical surgical resection with negative margins is the mainstay of treatment for vascular LMS [3]. While adjuvant radiation therapy could have a role in patients with primary liposarcoma, when concerning abdominal recurrence-free survival, no such effect was observed for leiomyosarcoma [6]. Studies are instead ongoing to evaluate neoadjuvant preoperative chemotherapy [7].

Complete en bloc gross tumor resection should be always aimed for, even with the sacrifice of infiltrated contiguous organs. Within the retroperitoneal sarcoma group, vascular LMS are a subtype characterized by more clearly defined borders and organs that are closely adjacent but not directly invaded by the tumor. In addition, they can potentially be preserved; however attention should be directed to achieving microscopically negative margins on the vein of origin [3].

Given the rarity of the disease, the published literature mainly consists of single institution retrospective studies, which are mostly focused on inferior vena cava (IVC) LMS [8,9]. Leiomyosarcoma of the renal vein is extremely rare [10,11]. A recent review of the literature documents only 67 cases of renal LMS, which were treated in the majority of the cases with nephrectomy (89.6 %) [12].

Open surgery is, historically, the chosen surgical approach for the treatment of vascular LMS, mainly due to the retroperitoneal location of these tumors. However, it is burdened with high postoperative morbidity. A minimally invasive approach, including laparoscopic and robotic surgery, even if is not the standard approach, could be pursued in selected cases instead [13,14]. A minimally invasive surgical approach of the retroperitoneum is currently routine in gyneco-oncological and urological surgery when aortic lymphadenectomy is performed, thus rendering the retroperitoneal site no longer an absolute limit to minimally invasive surgery [15,16]. In particular, robotic surgical systems, thanks to three-dimensional vision and enhanced ergonomics, have overcome the limits of traditional laparoscopy by facilitating the surgery of the great vessels.

In the literature, there are few cases of vascular LMS treated with robotic surgery. Saltzman et al. [17] described the first resection using a robotic laparoscopic approach for a primary leiomyosarcoma of the left renal vein. In this case, the vascular LMS was a 4.8 × 4.3-cm perihilar mass removed en bloc with the kidney and adrenal gland. One of the factors that mainly guides the choice of surgical approach is the size of the tumor [18]. Another case of the robotic approach in the context of vascular LMS was reported in 2018. This case documented a right perirenal tumor of almost 3 cm compatible with leiomyosarcoma arising from the right renal vein and was treated with a partial right vein resection and direct suture [13]. Our case is the only case reported in the literature regarding robotic renal vein resection for vascular LMS without associated nephrectomy or direct suture of the vessel. Ligation of the left renal vein with a preservation of the kidney and proximal collateral veins is widely described in abdominal aortic aneurysm surgery [19]. Due to the proximity to the inferior vena cava (IVC), as well as the presence of a large gonadal vein as collateral, the resection of the left venal rein was conducted with the preservation of the left kidney, thereby respecting the oncological criteria of vascular LMS treatment with negative margins. The size of the tumor was 4 × 2.2 × 2 cm, thus rendering feasible to perform a resection with the robotic approach.

Moreover, a possible limiting factor in the minimally invasive approach of vascular LMS is the need for vascular reconstructions after vascular resection. In the literature, as regarding robotic surgery, there is a lack of data; only a single case report of a retroperitoneal leiomyosarcoma robotic resection with an inferior vena cava graft replacement is available [20].

In the present case, thanks to the preoperative histological diagnosis and radiological study of the neoplasm, it was possible to proceed to a highly personalized and minimally invasive treatment. Following the most recent literature, the preoperative awareness that it was a vascular LMS, allowed surgery to be performed without the removal of the surrounding organs not directly involved. Additionally, the radiological evidence that the confluence with the left ovarian vein was not involved, allowed the renal vein to be resected, thus leaving an adequate venous outflow to the left kidney, which was then saved.

A consequence of the developments in surgical strategy, as well as the knowledge of different molecular pathways influencing oncological disease is that personalized surgery is now a reality. The forthcoming research trend is to achieve a more accurate, individualized approach. For these reasons, the technology is still evolving and minimally invasive surgery will continue to play a significant part in increasing oncological outcomes, as well as in reducing the impact of cancer treatments on the quality of life in selected patients.

## 4 Conclusions

In selected cases, a minimally invasive surgery of vascular leiomyosarcoma could be a feasible and safe treatment option when performed by experienced hands and with respect for the oncological criteria of VLMS treatment.

## Declaration of informed consent

Written consent for the publication of this case report and its accompanying images has been acquired from the patient. There is no information in submitted manuscript that can be used to identify the patient.

## Consent

Written informed consent was obtained from the patient for publication and any accompanying images. A copy of the written consent is available for review by the Editor-in-Chief of this journal on request.

## Ethical approval

Ethical clearance is not required for this case report, according to our institution's research ethics committee. The committee has verified that the report adheres to standard clinical practices and does not involve experimental interventions.

## Funding

The authors received no financial support for the preparation, research, authorship, and/or publication of this manuscript.

## Author contribution

Conceptualization: Claudio Lodoli, Miriam Attalla EL Halabieh.

Data Curation: Fabio Pacelli, Carlo Abatini.

Formal Analysis: Francesco Santullo, Carlo Abatini.

Funding Acquisition: Not applicable.

Investigation: Claudio Lodoli, Miriam Attalla EL Halabieh.

Project Administration: Claudio Lodoli, Valerio Gallotta.

Resources: Francesco Santullo, Fabio Pacelli.

Supervision: Fabio Pacelli, Valerio Gallotta.

Validation: Claudio Lodoli, Miriam Attalla EL Halabieh.

Visualization: Carlo Abatini, Miriam Attalla EL Halabieh

Writing-original draft preparation: Claudio Lodoli, Miriam Attalla EL Halabieh.

Writing-review and editing: Claudio Lodoli, Miriam Attalla EL Halabieh, Valerio Gallotta.

## Guarantor

Miriam Attalla El Halabieh.

## Research registration number

N/A.

## Conflict of interest statement

The authors do not have any potential conflicts of interest with respect to this manuscript.
